# What is the Optimal RSV Immunization Strategy for Infants Born in the RSV Off-season?

**DOI:** 10.1093/infdis/jiag287

**Published:** 2026-06-05

**Authors:** Hannah C Moore, Damien Foo, Fiona Giannini, Cara Minney-Smith, Christopher C Blyth, Peter C Richmond, Paul Effler, Mohinder Sarna, STAMP RSV Investigators, Hannah C Moore, Christopher C Blyth, Samantha Carlson, Fiona Giannini, Mohinder Sarna, David Foley, Catherine Hughes, Peter Richmond, Avram Levy, Ewan Cameron

**Affiliations:** Wesfarmers Centre for Vaccines and Infectious Diseases, The Kids Research Institute Australia, The University of Western Australia, Nedlands, Western Australia, Australia; School of Population Health, Curtin University, Bentley, Western Australia, Australia; Wesfarmers Centre for Vaccines and Infectious Diseases, The Kids Research Institute Australia, The University of Western Australia, Nedlands, Western Australia, Australia; Faculty of Health Sciences, Curtin University, Bentley, Western Australia, Australia; Wesfarmers Centre for Vaccines and Infectious Diseases, The Kids Research Institute Australia, The University of Western Australia, Nedlands, Western Australia, Australia; National Centre for Epidemiology and Population Health, The Australian National University, Canberra, Australian Capital Territory, Australia; PathWest Laboratory Medicine, QEII Medical Centre, Nedlands, Western Australia, Australia; Wesfarmers Centre for Vaccines and Infectious Diseases, The Kids Research Institute Australia, The University of Western Australia, Nedlands, Western Australia, Australia; PathWest Laboratory Medicine, QEII Medical Centre, Nedlands, Western Australia, Australia; Department of Infectious Diseases, Child and Adolescent Health Service, Perth Children’s Hospital, Nedlands, Western Australia, Australia; School of Medicine, University of Western Australia, Nedlands, Western Australia, Australia; Wesfarmers Centre for Vaccines and Infectious Diseases, The Kids Research Institute Australia, The University of Western Australia, Nedlands, Western Australia, Australia; School of Medicine, University of Western Australia, Nedlands, Western Australia, Australia; School of Medicine, University of Western Australia, Nedlands, Western Australia, Australia; Communicable Diseases Control Directorate, Western Australian Department of Health, Perth, Western Australia, Australia; Wesfarmers Centre for Vaccines and Infectious Diseases, The Kids Research Institute Australia, The University of Western Australia, Nedlands, Western Australia, Australia; School of Population Health, Curtin University, Bentley, Western Australia, Australia

**Keywords:** respiratory syncytial virus (RSV), immunization, seasonality, infant, hospitalization

## Abstract

We explored age-specific infant respiratory syncytial virus (RSV) laboratory-confirmed hospitalization rates by birth month in Western Australia. Population-based analyses were supplemented with susceptible-exposed-infected-recovered-(susceptible) mathematical model simulations. RSV-hospitalizations peaked for infants aged 1–3 months born in-season and 6–10 months born out-of-season, that is spring and summer-born infants. Offering an additional nirsevimab dose to spring and summer-born infants could avert significant hospitalizations and healthcare costs.

## Teaser

We used population-based analyses and model simulations to explore age-specific infant RSV-hospitalization rates by season of birth and showed that infants born out-of-season may benefit from a monoclonal antibody given around age 3 months, in addition to maternal vaccination.

Respiratory syncytial virus (RSV) is a major cause of acute lower respiratory infections globally, with a disproportionately high burden in young infants [1]. Fortunately, two passive immunization strategies are available to combat this high burden in infants: a vaccine administered during late pregnancy (Abrysvo®, RSVpreF), and a single dose long-acting monoclonal antibody (Beyfortus®, nirsevimab, and more recently Enflonsia®, clesrovimab). Following recent licensure of these immunization products, multiple countries have implemented immunization programs using either nirsevimab and/or RSVpreF.

In February 2025, Australia implemented a hybrid RSV Mother and Infant Protection Program, providing access to both RSVpreF and nirsevimab. In 2024, prior to the availability of RSVpreF, Western Australia (WA) implemented a nirsevimab only program. Currently, RSVpreF is offered year-round for all pregnancies through the National Immunization Program. Nirsevimab is funded by individual Australian States/Territories, but only for infants whose mothers did not receive RSVpreF, infants whose mothers were vaccinated <14 days before birth and infants at higher risk of severe RSV (preterm <32 weeks, congenital heart disease, immunosuppression, chronic lung disease, neurological conditions, cystic fibrosis, and Trisomy 21) [2]. Some Australian jurisdictions offer nirsevimab year-round, while others like WA offer nirsevimab in temperate areas of the state from April to September, coinciding with known seasonal activity [3] and circulating RSV patterns in the Southern Hemisphere. Real-world studies have shown RSVpreF and nirsevimab to be effective at preventing RSV-associated hospitalizations up to 6 months of age (71.3% for RSVpreF [4]) and 6 months post administration (88.2% for nirsevimab [5]) with the highest point estimates in the first 3 months from birth for RSVpreF, and since time of administration for nirsevimab. Real-world effectiveness data showing protection beyond age 6 months is lacking.

Given the limited duration of protection provided by both products and highly seasonal nature of RSV in most temperate regions, the optimal strategy to protect infants who are born out-of-season remains unclear. The aim of this study was to explore the age-specific RSV-hospitalization rates in infants up to the age of 12 months by seasons of birth in WA.

## METHODS

WA covers the western third of Australia and has a population of approximately 2.8 million. Over 95% of infants reside in regions where RSV is highly seasonal, peaking in winter. We used statewide, whole-of-population linked administrative health data from the Respiratory Infections Linked Data Platform [6] of births in WA between 1 January 2010 and 30 April 2024. Data sources included the Midwives Notification System comprising perinatal information on >99% of WA births, birth and death registries, the Hospital Morbidity Data Collection and routine microbiological testing from the state's sole pathology provider of public hospital patients, PathWest. Data were probabilistically linked through WA Data Services.

Our primary outcome was all-cause hospitalization temporally linked to laboratory-confirmed RSV, through polymerase chain reaction (herein named RSV-hospitalizations), with a specimen collection date within 4 days of hospital admission as previously defined [6], from 1 January 2010 to 30 April 2024, the last date of available data and only one month into the inaugural nirsevimab program in WA.

We calculated monthly age-specific incidence rates for RSV-hospitalizations in infants from 0 to 12 months using time-to-event Cox regression survival analysis techniques, allowing for multiple RSV-hospitalizations per infant as the numerator and person-time at-risk as the denominator. Infant-time at-risk was calculated from date of birth and censored at the earliest of: age 12 months, end of the study period, date of death, or date of each hospitalization, with a 14-day washout period between subsequent hospitalizations. We excluded births and admissions from 2020 to 2021 due to atypical RSV seasonality resulting from COVID-19-associated nonpharmaceutical interventions [7].

Monthly RSV-hospitalization rates, overall and by season of birth, were reported per 1000 infant-years with associated 95% confidence intervals (CIs). Season of birth was defined as summer (infants born December-February), autumn (born March-May), winter (born June-August), and spring (born September-November). To validate our analyses, we simulated age-specific hospitalizations from our established dynamic compartmental susceptible-exposed-infectious-recovered-susceptible (SEIRS) model calibrated to our population-based data [8]. Full details of the model structure, duration of protection from RSVpreF and nirsevimab based on clinical trial data, assumptions and fitting processes are detailed elsewhere [9], however, for this present analysis, we explored (1) the impact of a year-round RSVpreF strategy with 60% coverage on infant age-specific incidence rates and (2) the impact of year-round RSVpreF strategy with 60% coverage plus a nirsevimab dose with 60% coverage based on birth season. Due to the population-based nature of our compartmental model, the spring- and summer-born infants in this scenario given an additional dose of nirsevimab is irrespective of RSVpreF.

## RESULTS

There were 411 900 births and 5593 RSV-hospitalizations in infants across 12 years of the study period. Births were distributed across calendar years with 25.1% born in summer, 26.3% in autumn, 24.2% in winter and 24.4% in spring. Further characteristics of the cohort are shown in Supplementary Table 1. Overall, RSV-hospitalization rates peaked at 32.3/1000 infant-years in the second month of life and gradually declined to 6.2 per 1000 infant-years by the twelfth month of life (Figure 1*A*; Table 1).

**Figure 1.**
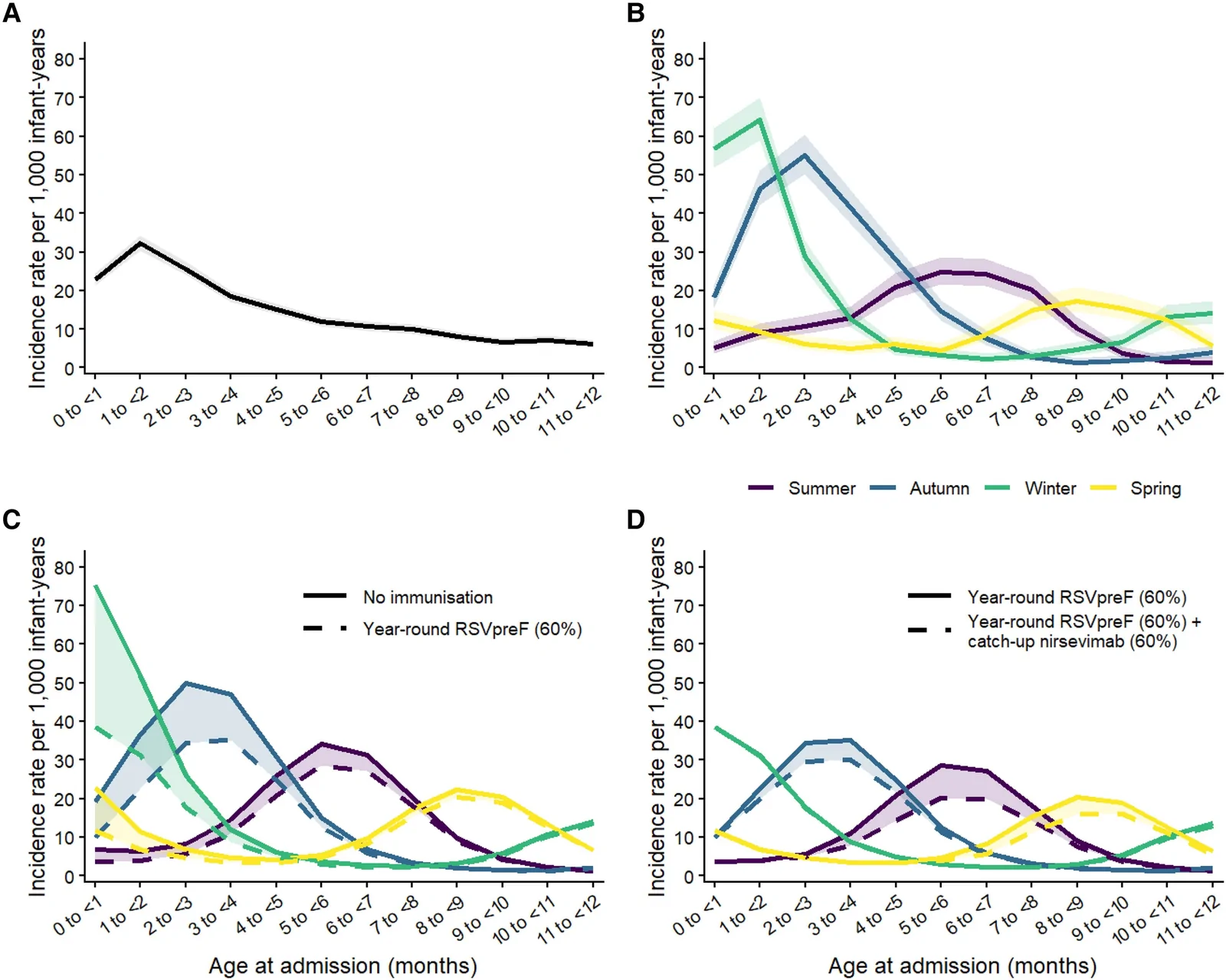
Monthly infant RSV-hospitalization rates in all births (Panel *A*), by infant season of birth (Panel *B*) January 2010-April 2024, simulated age-specific incidence rates by infant season of birth with no immunization, and with 60% coverage of year-round RSVpreF (maternal vaccine; Panel *C*), and simulated age-specific incidence rates by infant season of birth with 60% coverage of year-round RSVpreF (maternal vaccine) and 60% coverage of nirsevimab (Panel *D*). Note: Birth and admissions in 2020–2021 have been excluded. Shaded areas for Panels *A* and *B* represent 95% CIs. Shaded areas for Panel *C* represent the reduction from no immunization (solid line) to immunization (dashed line). Shaded areas for Panel *D* represent the reduction from maternal vaccination alone (solid line) to maternal vaccine and nirsevimab immunization (dashed line).

**Table 1.** Number and Incidence Rates of RSV-hospitalizations in Western Australia, Overall and by Season of Birth, 2010–2024

Age Group (months)	Number	Incidence Rate per 1000 Infant-years (95% CI)
Overall		
0 to <1	773	22.9 (21.3 to 24.6)
1 to <2	1076	32.3 (30.5 to 34.3)
2 to <3	839	25.6 (24 to 27.4)
3 to <4	594	18.4 (17.0 to 20.0)
4 to <5	482	15.2 (13.9 to 16.6)
5 to <6	372	11.9 (10.7 to 13.2)
6 to <7	330	10.7 (9.6 to 11.9)
7 to <8	302	9.9 (8.9 to 11.2)
8 to <9	243	8.1 (7.2 to 9.2)
9 to <10	196	6.7 (5.8 to 7.7)
10 to <11	210	7.2 (6.3 to 8.3)
11 to <12	176	6.2 (5.3 to 7.2)
Summer-born		
0 to <1	43	5.1 (3.8 to 7.0)
1 to <2	76	9.1 (7.3 to 11.5)
2 to <3	89	10.9 (8.9 to 13.5)
3 to <4	103	12.9 (10.7 to 15.8)
4 to <5	163	21.0 (18.1 to 24.6)
5 to <6	190	24.8 (21.6 to 28.7)
6 to <7	186	24.3 (21.1 to 28.2)
7 to <8	154	20.2 (17.3 to 23.7)
8 to <9	79	10.4 (8.3 to 13.1)
9 to <10	28	3.7 (2.6 to 5.5)
10 to <11	12	1.6 (0.9 to 3.0)
11 to <12	10	1.4 (0.8 to 2.8)
Autumn-born		
0 to <1	161	18.2 (15.7 to 21.4)
1 to <2	399	46.4 (42.1 to 51.3)
2 to <3	465	55.0 (50.3 to 60.3)
3 to <4	349	41.4 (37.4 to 46.1)
4 to <5	236	28.1 (24.8 to 32.0)
5 to <6	123	14.7 (12.3 to 17.6)
6 to <7	64	7.6 (6 to 9.9)
7 to <8	22	2.7 (1.8 to 4.2)
8 to <9	10	1.2 (0.7 to 2.5)
9 to <10	15	1.9 (1.2 to 3.4)
10 to <11	20	2.6 (1.7 to 4.2)
11 to <12	30	4.0 (2.8 to 5.8)
Winter-born		
0 to <1	467	56.7 (51.8 to 62.1)
1 to <2	525	64.1 (58.9 to 69.9)
2 to <3	236	28.9 (25.5 to 33.0)
3 to <4	104	12.8 (10.6 to 15.6)
4 to <5	37	4.6 (3.4 to 6.5)
5 to <6	25	3.2 (2.2 to 4.9)
6 to <7	17	2.2 (1.4 to 3.8)
7 to <8	22	3.0 (2.0 to 4.6)
8 to <9	34	4.6 (3.3 to 6.6)
9 to <10	47	6.6 (5.0 to 8.9)
10 to <11	92	13.3 (10.9 to 16.4)
11 to <12	96	14.1 (11.6 to 17.3)
Spring-born		
0 to <1	102	12.3 (10.1 to 15.0)
1 to <2	76	9.3 (7.5 to 11.7)
2 to <3	49	6.2 (4.7 to 8.3)
3 to <4	38	4.9 (3.6 to 6.9)
4 to <5	46	6.1 (4.6 to 8.2)
5 to <6	34	4.5 (3.3 to 6.5)
6 to <7	63	8.7 (6.8 to 11.2)
7 to <8	104	14.8 (12.2 to 18.0)
8 to <9	120	17.3 (14.5 to 20.8)
9 to <10	106	15.3 (12.7 to 18.6)
10 to <11	86	12.4 (10.1 to 15.5)
11 to <12	40	5.8 (4.3 to 8.0)

Births and admissions in 2020–2021 have been excluded. Summer-born equates to infants born December-February; Autumn-born equates to infants born March-May; Winter-born equates to infants born June-August; Spring-born equates to infants born September-November.

The high incidence of RSV-hospitalizations in early life was particularly prominent among autumn-and winter-born infants with RSV-hospitalization rates peaking for autumn-born infants aged 2–3 months at 55.0/1000 infant-years and for winter-born infants aged 1–2 months at 64.2/1000 infant-years. However, for summer- and spring-born infants, the distribution of age-specific RSV-hospitalizations was remarkably different. For summer-born infants, RSV-hospitalizations peaked during their fifth month of life at 21.0/1000 infant-years, sixth month of life at 24.8/1000 infant-years, followed by 24.3/1000 infant-years during their seventh month of life (Figure 1*B*; Table 1). This equates to an annual average of 43 RSV-hospitalizations for summer-born infants aged 5–8 months, or 47% of all RSV-hospitalizations in the first year of life for summer-born infants. For spring-born infants, RSV-hospitalizations, peaked at 8–10 months with rates at 14.8/1000 infant-years during their eighth month of life, 17.3/1000 infant-years in their ninth month and 15.3/1000 infant-years in their tenth month of life (Figure 1*B*; Table 1). This equates to an annual average of 27 RSV-hospitalizations for spring-born infants aged 8–10 months, or 38% of all RSV-hospitalizations for infants born in spring.

Our simulated modeling analyses with no immunization strategy replicated the age pattern for different seasons of birth. Implementing a year-round RSVpreF strategy with 60% coverage reduced the incidence in peak monthly age brackets for winter- and autumn-born infants but had little impact on summer- and spring-born infants (Figure 1*C*). For example, the simulated RSV incidence for winter-born infants at their peak in the first month of life reduced from 75.4/1000 infant-years to 38.6/1000; for autumn-born births at 2–3 months simulated RSV incidence reduced from 50.0/1000 to 34.5/1000; but for summer-born infants at their peak at 5–6 months, incidence only reduced from 34.1/1000 to 28.6/1000, and spring-born infants at their peak of 8–9 months from 22.4/1000 to 20.4/1000. However, adding a nirsevimab infant dose for summer- and spring-born infants with 60% coverage along with year-round RSVpreF with 60% coverage had an impact, particularly on summer-born infants (eg, simulated RSV incidence for summer-born infants at their highest peak [5–6 months] reduced from 28.6/1000 to 20.1/1000 infant-years) and spring-born infants (eg, simulated RSV incidence for spring-born infants at 8–9 months from 20.4/1000 to 16.1/1000; Figure 1*D*). This shows an absolute rate reduction of 8.5/1000 infant-years for summer-born infants at their peak compared to a 5.5/1000 rate reduction with only year-round RSVpreF at 60% coverage. Equivalent absolute rate reductions for spring-born infants are 1.9/1000 with only year-round RSVpreF compared to 4.3/1000 with an additional dose of nirsevimab.

## DISCUSSION

Our analysis of over 12 years of population-based data shows that summer-born infants in temperate WA experience their highest RSV-hospitalization rates between the ages of 5 and 8 months and spring-born infants between 8 and 10 months of age. This is likely beyond the period of protection provided by the maternal vaccine, RSVpreF that has, thus far, not shown protection lasting beyond 6 months of age. These findings are important for determining the optimal protection strategy for preventing RSV-hospitalizations in all infants. We acknowledge from clinical trial data, that protection from a maternal vaccine does not immediately cease at 6 months. Indeed, our SEIRS model represents the waning protection of RSVpreF from birth to 360 days based on clinical trial estimates [9]. However, while a maternal vaccine strategy providing protection for up to 6 months of age—with the highest protection from birth to age 3 months, as supported by observational effectiveness data—is optimal for infants born at the beginning or during the annual RSV season, it may leave an unprotected window for summer- and spring-born infants.

As nirsevimab can be administered to infants at any age up to 24 months and provides 6 months of protection, the optimal strategy to cover the peak ages of RSV-hospitalization for spring- and summer-born infants could be to offer nirsevimab prior to the onset of the RSV season when summer-born infants approach 3 months of age and spring-born infants approach 5 months of age. The 2024 WA nirsevimab program achieved 71% coverage among eligible infants and reduced RSV-hospitalizations by 57% [10]. Using those figures, we estimate that offering nirsevimab to spring- and summer-born infants in WA could avert an average of 65 additional infant RSV-hospitalizations annually. With an estimated index RSV-hospitalization cost for infants of AUD$12 643 (standard deviation $21 644) [11], this could translate into saving up to an additional AUD$821 795 in hospital costs per year. Our conclusions are supported by our simulated modeling analyses. Comprehensive details of our model fitting, calibration and assumptions are presented elsewhere [9], and here have shown that an additional nirsevimab dose for spring- and summer-born infants to a 60% coverage of year-round RSVpreF could reduce RSV-hospitalization incidence at their peak months by 8.5/1000 infant-years for summer-born infants and 4.3/1000 for spring-born infants (absolute rate reduction).

Both infant age and season of birth are known to be risk factors for infant RSV-hospitalizations [12]. An immunization strategy for a pathogen like RSV that is highly seasonal and for which incidence varies with age creates complexities for implementation. Optimal immunization strategies also need to be weighed up against logistical challenges of administrating a hybrid program with seasonal components, the cost of a vaccine versus a monoclonal antibody, and parental choice, which some Australian data suggest a preference for maternal vaccination [13].

The generalizability of our findings extends beyond WA. The overall age distribution of RSV-hospitalizations in our population mirrors global data from higher and upper-middle income countries [14]. This recent global analysis by chronological month of age and birth month highlighted that the risk of RSV-hospitalizations can vary substantially by month of birth, underscoring the importance of identifying the highest risk months when attempting to design an optimal immunization program for preventing severe infant RSV [14]. However, we do acknowledge our findings are most applicable to temperate settings.

Despite using population-based data, we were limited to public microbiology testing data, and from our previous work, we know RSV testing at public pathology providers declined between 2012 and 2018, especially in metropolitan areas of WA, and that RSV testing is higher in younger infants and those born preterm [15]. However, we do not believe the limitation of potential reductions in RSV testing, resulting in a lower number of RSV-confirmed hospitalizations in our analytic cohort, would translate into negating the seasonal pattern of RSV-hospitalizations by month of birth, which is the primary finding of this analysis.

In summary, we have shown through population-based analyses and model simulations that infants born out-of-season experience their highest hospitalization rates for RSV between the ages of 6 and 10 months; beyond the possible reach of protection of a maternal vaccine. These findings highlight a possible gap in protection that may be relevant when considering complementary infant immunization strategies with nirsevimab. In our context, offering nirsevimab to spring-summer born infants could potentially avert significant hospitalizations and healthcare costs, although the magnitude of this impact will depend on implementation and real-world effectiveness. In light of these findings, the WA Government has recently expanded eligibility for nirsevimab for infants born out-of-season, regardless of maternal vaccination status.

## Supplementary Material

jiag287_Supplementary_Data

## Supplementary Data

Supplementary materials are available at *The Journal of Infectious Diseases* online (http://jid.oxfordjournals.org/). Supplementary materials consist of data provided by the author that are published to benefit the reader. The posted materials are not copyedited. The contents of all supplementary data are the sole responsibility of the authors. Questions or messages regarding errors should be addressed to the author.
