# Supplementary material for: What is the Optimal RSV Immunization Strategy for Infants Born in the RSV Off-season?
Source: J Infect Dis. 2026 Jun 5;234(4):e823–8. doi: 10.1093/infdis/jiag287 (PMC13668356; doi:10.1093/infdis/jiag287)
Supplement: Supplementary file 1 [file jiag287_supplementary_data.docx]

**What is the optimal RSV immunisation strategy for infants born in the RSV off-season?**

Hannah C Moore, Damien Foo, Fiona Giannini, Cara Minney-Smith, Christopher C Blyth, Peter C Richmond, Paul Effler, Mohinder Sarna on behalf of the STAMP RSV Investigators

**Supplementary Material**

**Table S1.** Characteristics of study cohort (*N* = 411,900).

| **Characteristic** | **n (%)** |
| --- | --- |
| ***Sex*** |  |
| Female | 200,257 (48.6) |
| Male | 211,574 (41.4) |
| Indeterminate | 66 (0.0) |
| Missing | 3 (0.0) |
| ***First Nations status*** |  |
| First Nations | 28,110 (6.8) |
| Non First Nations | 383,785 (93.2) |
| Missing | 5 (0.0) |
| ***Preterm birth*** |  |
| Term birth (≥ 37 weeks) | 337,768 (82.0) |
| Moderate to late preterm birth (32 to ≤ 36 weeks) | 28,463 (6.9) |
| Very preterm birth (28 to ≤ 31 weeks) | 2,822 (0.7) |
| Extremely preterm birth (< 28 weeks) | 2,966 (0.7) |
| Missing | 39,881 (9.7) |
| ***Remoteness area at birth^a^*** |  |
| Major Cities of Australia | 271,488 (65.9) |
| Inner Regional Australia | 26,178 (6.4) |
| Outer Regional Australia | 26,117 (6.3) |
| Remote Australia | 13,259 (3.2) |
| Very Remote Australia | 9,852 (2.4) |
| Missing | 65,006 (15.8) |
| ***Socioeconomic status at birth ^b^*** |  |
| Quintile 1 (most disadvantaged) | 11,091 (2.7) |
| Quintile 2 | 38,947 (9.5) |
| Quintile 3 | 53,092 (12.9) |
| Quintile 4 | 144,367 (35.1) |
| Quintile 5 (least disadvantaged) | 121,798 (29.6) |
| Missing | 42,605 (10.3) |
| ***Season of birth*** |  |
| Summer (born December-February) | 103,283 (25.1) |
| Autumn (born March-May) | 108,155 (26.3) |
| Winter (born June-August) | 99,834 (24.2) |
| Spring (born September-November) | 100,628 (24.4) |
| ***Year of birth*** |  |
| 2010 | 31,270 (7.6) |
| 2011 | 32,210 (7.8) |
| 2012 | 33,874 (8.2) |
| 2013 | 34,411 (8.4) |
| 2014 | 35,220 (8.6) |
| 2015 | 35,001 (8.5) |
| 2016 | 35,895 (8.7) |
| 2017 | 34,563 (8.4) |
| 2018 | 33,441 (8.1) |
| 2019 | 33,377 (8.1) |
| 2022 | 32,620 (7.9) |
| 2023 | 30,024 (7.3) |
| 2024^c^ | 9,994 (2.4) |

Note: Births and admissions in 2020-2021 have been excluded. Summer-born equates to infants born December-February; Autumn-born equates to infants born March-May; Winter-born equates to infants born June-August; Spring-born equates to infants born September-November.

^a^ Remoteness area was based on the Accessibility/Remoteness Index of Australia developed by the Australian Bureau of Statistics.

^b^ Socioeconomic status was based on the Socioeconomic Index for Areas measure of relative socioeconomic advantage and disadvantage developed by the Australian Bureau of Statistics.

^c^ We included births up to 30 April 2024 due to data availability of hospital data.

**References**

1. Australian Bureau of Statistics. Remoteness Areas: Australian Statistical Geography Standard (ASGS) Edition 3. 2023. Available from: <https://www.abs.gov.au/statistics/standards/australian-statistical-geography-standard-asgs-edition-3/jul2021-jun2026/remoteness-structure/remoteness-areas>.
2. Australian Bureau of Statistics. Socio-Economic Indexes for Areas (SEIFA), Australia. 2023. Available from <https://www.abs.gov.au/statistics/people/people-and-communities/socio-economic-indexes-areas-seifa-australia/latest-release>.
